# Marburg Virus Infection Detected in a Common African Bat

**DOI:** 10.1371/journal.pone.0000764

**Published:** 2007-08-22

**Authors:** Jonathan S. Towner, Xavier Pourrut, César G. Albariño, Chimène Nze Nkogue, Brian H. Bird, Gilda Grard, Thomas G. Ksiazek, Jean-Paul Gonzalez, Stuart T. Nichol, Eric M. Leroy

**Affiliations:** 1 Centers for Disease Control and Prevention, Special Pathogens Branch, Atlanta, Georgia, United States of America; 2 Centre International de Recherches Médicales de Franceville, Franceville, Gabon; 3 Institut de Recherche pour le Développement, UR178, Franceville, Gabon; 4 School of Veterinary Medicine, University of California at Davis, Davis, California, United States of America; 5 Institut de Recherche pour le Développement, UR178, Nakhonpathom, Thaïland; Cambridge University, United Kingdom

## Abstract

Marburg and Ebola viruses can cause large hemorrhagic fever (HF) outbreaks with high case fatality (80–90%) in human and great apes. Identification of the natural reservoir of these viruses is one of the most important topics in this field and a fundamental key to understanding their natural history. Despite the discovery of this virus family almost 40 years ago, the search for the natural reservoir of these lethal pathogens remains an enigma despite numerous ecological studies. Here, we report the discovery of Marburg virus in a common species of fruit bat (*Rousettus aegyptiacus*) in Gabon as shown by finding virus-specific RNA and IgG antibody in individual bats. These Marburg virus positive bats represent the first naturally infected non-primate animals identified. Furthermore, this is the first report of Marburg virus being present in this area of Africa, thus extending the known range of the virus. These data imply that more areas are at risk for MHF outbreaks than previously realized and correspond well with a recently published report in which three species of fruit bats were demonstrated to be likely reservoirs for Ebola virus.

## Introduction

Forty years after the discovery of Marburg virus as the cause of a hemorrhagic fever outbreak among laboratory workers in Germany, the natural reservoir for this highly pathogenic filovirus remains unknown [1]. Until 2000, the virus origins of all Marburg hemorrhagic fever (MHF) cases could be traced to eastern Africa. However, in 2005 the largest outbreak of MHF on record occurred in Uige, Angola, expanding the known range of the disease, and likely the natural reservoir, to the far western edge of the Congo basin [2], [3]. We hypothesized that Marburg virus is present in the rain forests of Gabon, based on ecologic similarities and relative proximity (<800 km) to northern Angola. Bats were the focus of this study based on the recent discovery of the related filovirus, Ebola virus, in fruit bats in Gabon and Republic of Congo [4] (RC), and epidemiologic linkage of MHF cases to a gold mine containing sizeable numbers of bats during a large MHF outbreak in Durba, Democratic Republic of Congo (DRC) in 2000 [5], [6]. Further evidence for a bat reservoir include the linkage of a MHF case in 1987 to Kitum cave at Mt. Elgon, Kenya [7], and transient viremias in asymptomatic bats experimentally infected with Ebola virus [8].

Here, we report testing of bats collected from Gabon and Republic of Congo and demonstrate Marburg virus infection in a common species of fruit bat (*Rousettus aegyptiacus*) as evidenced by the presence of virus specific RNA and antibody.

## Materials and Methods

### Marburg virus nucleic acid and IgG detection

For each bat, approximately 100 mg of tissue was incubated overnight at 4°C in 450 ul of cold 2X cellular lysis buffer (ABI) to inactivate virus. Each tissue was then diluted to 1X and homogenized for 2 minutes, at 1500 strokes/min using a ball-mill tissue grinder (Genogrinder 2000, Spex Centriprep). Total RNA was extracted from ∼150 ul of the homogenate [9] and tested for Marburg virus using slightly modified real-time [10] or nested RT-PCR assays. The nested VP35 RT-PCR assay is previously described [6], while the four primers used for the nested NP assay are (5′ to 3′) MBG704F1-GTAAAYTTGGTGACAGGTCATG, MBG719F2-GGTCATGATGCCTATGACAGTATCAT, MBG1248R1-TCTCGTTTCTGGCTGAGG, and MBG1230R2-ACGGCIAGTGTCTGACTGTGTG. The annealing conditions were 50° C for the first round and 55° C for the second round using high-fidelity RT-PCR reagents (Invitrogen). Primer concentrations and amplification conditions used were as described by the manufacturer. Samples 1448, 1519, 1631 and 2296 were independently assayed a minimum of three times in the real-time RT-PCR assay, each time using RNA extracted from newly cut tissue. Potential false positives due to PCR or sample cross-contamination could be ruled out due to the unique virus sequences obtained and the complete lack of any previous Marburg virus testing in the laboratory. The positive control RNA used for the PCR analysis was derived from the Ravn 1987 isolate and is >15% divergent from the known sequence obtained from the three nested RT-PCR-positive bats.

IgG was detected from bat sera diluted 1∶100 using a previously described protocol modified for Marburg virus [11]. Bats with corrected OD values >0.13 were additionally tested at 1∶400 and 1∶1600 dilutions. Bat IgG was detected using Protein A-G peroxidase or HRP-conjugated goat sera raised against a cocktail of IgG from six diverse bat species. Bats of the species *Epomops franqueti* (N = 47) were used as the negative control group.

### Phylogenetic analyses

Bat derived sequence fragments (Genbank accession numbers EU068108-13) were concatenated then aligned with 18 MBG virus genomes (Genbank accession numbers DQ447649-60, AY358025, DQ217792, Z29337.) Maximum likelihood analyses (bootstrap 500 replicates) were completed (PAUP v4.0b10, Sinauer.). Genetic distances were calculated using the Wisconsin package of GCG version 10.3. (Accelrys Inc., San Diego, CA).

### Collection of bat organs

Bat species were morphometrically determined in the field using the species identification key developed by Bergman [12]. In addition, bats were photographed and catalogued noting weight, sex, age (adult or juvenile) and forearm measurements. Bats were euthanized individually after which the organs were immediately harvested and frozen in liquid nitrogen. All organs were then stored under nitrogen vapor until placed in a −80 mechanical freezer for storage for subsequent analysis.

## Results and Discussion

**Figure 1:**
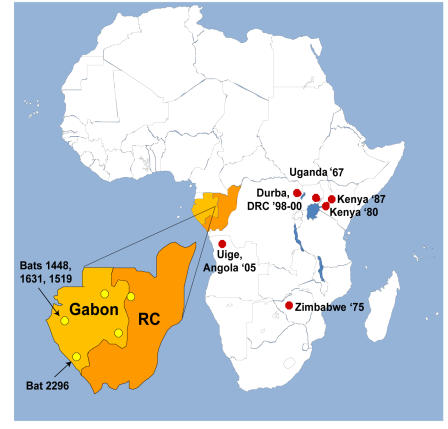
Animal collection sites in Gabon and Republic of Congo. Animal trap locations in Gabon and Republic of Congo (expansion) are indicated by yellow circles. Also indicated are the locations of the four PCR positive bats (by arrows) and the dates and locations of all known origins of previous Marburg virus outbreaks (red circles).

We tested over 1100 bats representing 10 species (Table 1) collected from five locations throughout Gabon and northwest Republic of Congo (Figure 1), and show evidence of Marburg virus infection in only one species, *Rousettus aegyptiacus*. Homogenized liver and spleen samples of 1138 bats were first analyzed using a Marburg virus-specific real-time RT-PCR assay to the VP40 gene recently used for human diagnostic testing during the 2005 Angola MHF outbreak [10]. Four bats (sample numbers 1448, 1631, 2296 and 1519), all *Rousettus aegyptiacus*, were positive at low levels (cycle threshold values >33) in the real-time assay. All four of these bats were trapped near caves in 2005 and early 2006 at two geographic locations in Gabon 250 km apart and approximately 700 km north of Uige, Angola. These four samples were then subjected to further analysis by conventional nested RT-PCR targeting the virus VP35 and NP genes (Materials and Methods). Three bats tested positive in each of the nested assays while a fourth bat (sample 1519), though never found positive by conventional RT-PCR, tested positive five independent times in the real-time assay, each time using RNA extracted from newly cut tissue.

**Table 1:** Summary of bat species tested for Marburg virus specific RNA and/or antibody.

Species	No. In collection	PCR testing			Marburg IgG testing	
		Tested	Positive by Q-RT-PCR	Positive by nested-PCR	Tested	Corrected OD>0.13
*Megaloglossus woermanni*	37	37	0	0	20	0
*Micropteropus pusillus*	149	149	0	0	19	0
*Hypsignathus monstrosus ^‡^*	57	56	0	0	12	0
*Epomops franqueti ^‡^*	296	296	0	0	47	0
*Hipposideros gigas*	1	1	0	0	1	0
*Rousettus aegyptiacus*	285	283	4	3	242	29
*Myonicterus torquata ^‡^*	264	264	0	0	55	0
*Casinycteris argynnis*	2	2	0	0	0	0
*Eidolon helvum*	36	35	0	0	33	0
Microchiroptere *	15	15	0	0	9	0
*Total*	1142	1138	4	3	438	29

Description of bat species and their respective numbers analyzed by either PCR or serology followed by the number of bats of each respective species that tested positive by real-time RT-PCR, nested RT-PCR or had corrected OD values greater than 0.13 at serum dilutions of 1∶100. (*) denotes unspeciated bats of the suborder Microchiroptera. (‡) denotes bat species previously identified to show evidence of Ebola infection [4].

**Figure 2:**
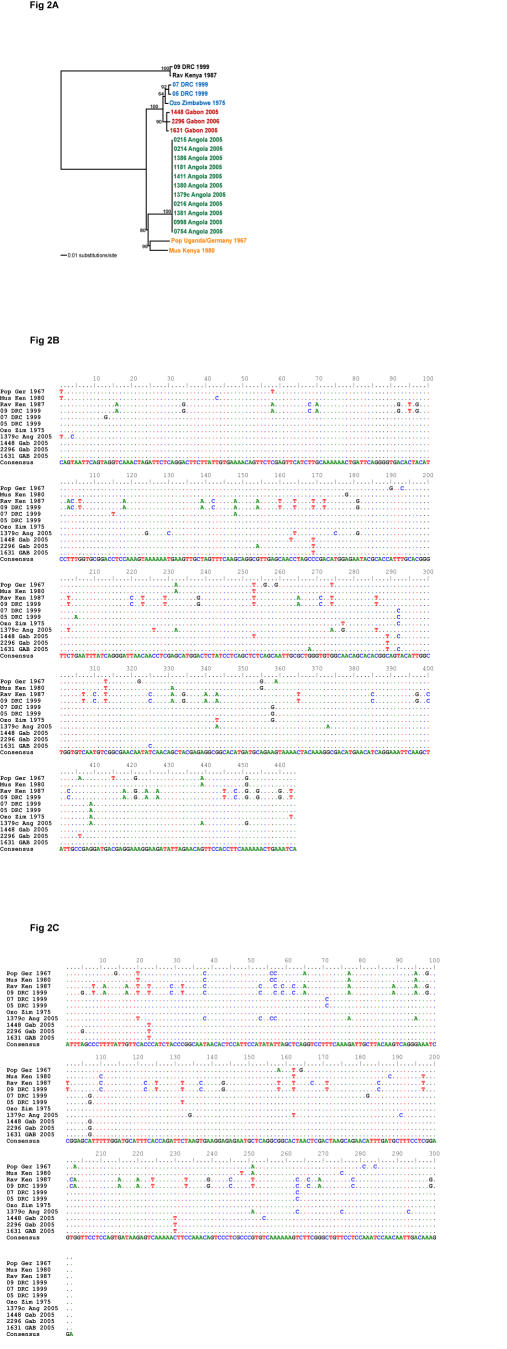
Phylogenetic analyses and nucleotide sequence alignments of NP and VP35 sequences derived from bat tissues. A) Maximum likelihood analysis of the concatenated NP (464 nt) and VP35 (302 nt) sequence fragments obtained from each bat specimen and 18 MBG virus isolates. Bootstrap support values are indicated at the nodes. Abbreviations of historical isolates are Rav = Ravn, Ozo = Ozolin, Pop = Popp and Mus = Musoke. B-C) Nucleotide alignment of the sequences in (A) in which the lineages from the Angola 2005 outbreak are singly represented by Ang1379c.

Sequence analysis of the purified PCR products identified unique sequences from each bat which together form a well supported single lineage distinct from all previously characterized Marburg viruses, including the Ravn strain used as a positive control in these PCR-based assays (Figure 2A). The Gabon bat Marburg virus sequences differed from those of the other western African Marburg virus lineage, Angola, by approximately 5% at the nucleotide level, far less than the 15% diversity observed among East African Marburg virus isolates (data not shown). An alignment of the NP and VP35 sequences (Figures 2B and C) shows the three bat sequences are divergent from each other at nine positions, and different from a consensus sequence of eight historical Marburg virus isolates at 14 positions. We suspect that the virus load of the fourth bat (positive in the real-time assay only) is just below the limit of detection by the nested assays and/or the sequence has mis-matches at critical PCR-priming positions in the NP and VP35 assays.

**Figure 3:**
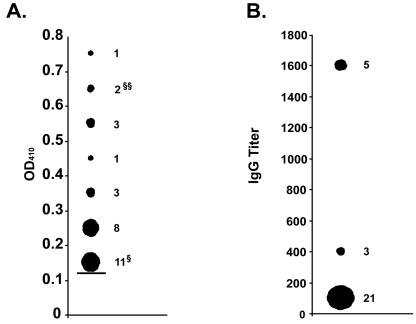
Marburg antibody testing in bats collected at locations where PCR positive bats were found. A.) Corrected OD values from bat sera diluted 1∶100 that are greater than the threshold value of 0.13 (solid horizontal bar) which was calculated as the average corrected OD of the negative control group (*E. franqueti*, N = 47) plus 3 standard deviations. The numbers of sera specimens used to calculate the values are shown to the right of the corresponding symbol. OD values from nested-PCR positive bats 1448, 2296 (§§) and 1631 (§) are also noted. B.) Antibody titers of sera specimens with corrected OD values greater than 0.13. The numbers of serum specimens used to calculate the values are shown to the right of the corresponding symbol.

After screening the collection of bats by real-time RT-PCR, we tested for Marburg virus specific antibody in sera (if available) from all bats trapped at the two locations (438 bats) from which PCR positive bats were found (Table 1). Interestingly, sera from 29 bats had corrected OD values greater than 0.13, a threshold value that is three standard deviations from the average OD of the control group (*Epomops franqueti*) (Figure 3A). Moreover, all 29 of these bats were *Rousettus aegyptiacus* while none of the other species tested (N = 196) had corrected OD values greater than this threshold value. Sera from three of the 29 bats had OD values greater than 0.13 when diluted 1∶400 while another five bats, including 1448 and 2296, met the same criteria at dilutions of 1∶1600 (Figure 3B). Unfortunately, serum was unavailable from bat 1519 that had tested positive by real-time RT-PCR. Initial serologic testing was completed with a protein A/G conjugate. In the event that the protein A/G conjugate showed a species-specific preference for *Rousettus aegyptiacus* IgG, we re-tested those sera with OD values greater than 0.13 (in addition to 50 Marburg antibody “negative” bats) using a goat anti-bat conjugate made by immunization with IgG from multiple diverse bat species (both micro and mega-chiropterans). The results of this secondary testing were identical to the initial serological findings using protein A/G (data not shown). These data indicate a substantial fraction, almost 9%, of *Rousettus aegyptiacus* trapped at these locations may have low-level antibody to Marburg virus, while another 3% have more significant Marburg antibody titers. Among the *R. aegyptiacus* population tested for which age determinations could be made, evidence of Marburg infection in bats favored adults (24/138) over juveniles (4/86), 17.4% to 4.6% respectively (p = 0.005, Chi-Square test ). However, firm conclusions about the proportion of infected adult versus juvenile populations are difficult because the majority of ‘positive’ bats show only low titers of Marburg antibody while those bats with more conclusive evidence of Marburg infection, by being either PCR positive and/or having IgG titers greater than or equal to 1∶400 (N = 8), are more equivalently distributed (five adults and three juveniles). In addition, there could be residual maternal Marburg-specific antibody in the juvenile bat population. Fifteen of the bats, with OD values greater than 0.13, were males while among the three PCR-positive animals, two were adults (male and female) and one was a juvenile (male).

The serological data, combined with the PCR data, are suggestive that these bats may represent a bon-a-fide reservoir species. However, we cannot rule out periodic contact by the bats with an as yet unnamed reservoir. The presence of both virus RNA and IgG antibodies in three (or possibly four) of the animals is consistent with extended viremias, but may well represent late acute phase infections. While it is difficult to determine from these data if Marburg virus causes significant morbidity in *R. aegyptiacus*, it is worth noting that all of the animals caught appeared clinically healthy and were strong enough to leave their roost to forage for food. Virus isolation attempts and antigen detection tests on the same liver/spleen organ extracts were negative, which along with the quantitative PCR data, indicate low levels of Marburg virus in these organs.

**Figure 4:**
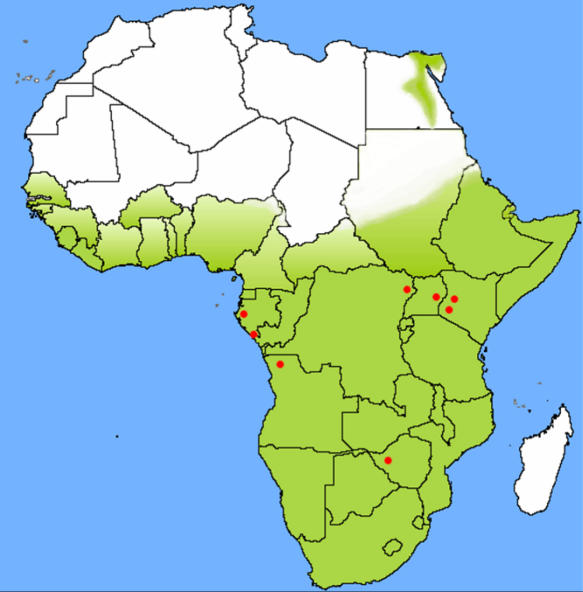
Geographic distribution (green shade) of *Rousettus aegyptiacus* in Africa. Red dots indicate the locations of all previous known Marburg virus outbreaks, as indicated in Figure 1, in addition to the locations of the Marburg-positive bats.

Although inconclusive, several other lines of evidence are consistent with *R. aegyptiacus* representing a natural host for Marburg virus. Cave roosting is not generally observed with most fruit bats [13], including the three species thought to harbor Ebola virus [4]. However, *R. aegyptiacus* is known to roost in caves, a behavior that correlates well with the epidemiologic linkage of greater than 80% of human cases in the Durba MHF outbreak to mining activity in a gold mine [5] harboring large bat populations. Furthermore, the home range of *R. aegyptiacus* encompasses the geographic origin of all known sources of Marburg virus outbreaks (Figure 4) [13] as well as the locations from which the Marburg virus PCR and IgG positive bats were found. These Marburg virus positive bats represent the first naturally infected non-primate animals ever identified, and this is the first report of any Marburg virus activity in Gabon, a region of Africa recently hit by multiple outbreaks of the highly pathogenic Ebola virus (species Zaire) [14]. Together, these facts predict that 1) the potential for Marburg virus contact could be more wide-spread than previously recognized, 2) fruit bats from the sub-family *Pteropodinae* may serve as filovirus hosts and 3) the species of fruit bats that harbor Ebola and Marburg viruses are likely distinct, yet their home ranges may have large areas of overlap. Identification of the reservoir host should allow development of risk reduction measures to help mitigate the potential of future disease outbreaks.

## References

[1] Sanchez A, Geisbert TW, Feldmann H, Knipe DM, Howley PM (2007). Filoviruses.. Fields Virology..

[2] World Health Organization (2005). Marburg virus disease, Angola.. Wkly Epidemiol Rec.

[3] Centers for Disease Control and Prevention (2005). Outbreak of Marburg virus hemorrhagic fever-Angola, October 1, 2004–March 29.. Morb Mortal Wkly Rep.

[4] Leroy EM, Kumulungui B, Pourrut X, Rouquet P, Hassanin A (2005). Fruit bats as reservoirs of Ebola virus.. Nature.

[5] Bausch DG, Borchert M, Grein T, Roth C, Swanepoel R (2003). Risk Factors for Marburg Hemorrhagic Fever, Democratic Republic of the Congo.. Emerg Infect Dis.

[6] Bausch DG, Nichol ST, Muyembe-Tamfum J-J, Borchert M, Rollin PE, Bausch DG (2006). Marburg hemorrhagic fever associated with multiple genetic lineages of virus.. N Engl J Med.

[7] Johnson ED, Johnson BK, Silverstein D, Tukei P, Geisbert TW (1996). Characterization of a new Marburg virus isolated from a 1987 fatal case in Kenya.. Archives of Virology.

[8] Swanepoel R, Leman PA, Burt FJ (1996). Experimental inoculation of plants and animals with Ebola virus.. Emerg Infect Dis.

[9] Towner JS, Sealy TK, Ksiazek TG, Nichol ST (2007). High-throughput molecular detection of hemorrhagic fever virus threats with applications for outbreak settings.. J Infect Dis.

[10] Towner JS, Khristova ML, Sealy TK, Vincent MJ, Erickson BR (2006). Marburgvirus genomics and association with a large hemorrhagic fever outbreak in Angola.. J Virol.

[11] Ksiazek TG, West CP, Rollin PE, Jahrling PB, Peters CJ (1999). ELISA for the detection of antibodies to Ebola viruses.. J Infect Dis.

[12] Bergmans W (1989). Taxonomy and biogeography of african fruit bats (Mammalia, Megachiroptera).. Beaufortia.

[13] Kingdon J (1974). East African Mammals..

[14] Leroy EM, Rouquet P, Formenty P, Souquière S, Kilbourne A (2004). et al.. Science.

